# Morbid Obesity and Severe Knee Osteoarthritis: Which Should Be Treated First?

**DOI:** 10.1007/s11605-022-05272-6

**Published:** 2022-02-24

**Authors:** Stephanie Purcell, Intekhab Hossain, Bradley Evans, Geoff Porter, Glen Richardson, James Ellsmere

**Affiliations:** 1 Department of Surgery, Dalhousie University, 1276 South Park, Vic 823, Halifax, NS B3H 2Y9 Canada; 2 Department of Surgery, Memorial University, St. John’s, Newfoundland and Labrador Canada

**Keywords:** Morbid obesity, Bariatric surgery, Sleeve gastrectomy, Osteoarthritis, Total knee arthroplasty, Complications

## Abstract

**Background:**

There are limited prospective data, and conflicting retrospective data, providing guidance on how to optimally manage patients with morbid obesity and severe knee osteoarthritis. This study sought to review the effect of bariatric surgery on knee pain and knee surgery 30-day outcomes, as well as assess whether the sequence of bariatric and knee surgery has any effect on 30-day complications.

**Methods:**

A retrospective chart review of all patients undergoing laparoscopic sleeve gastrectomy (LSG) from July 2006 to July 2016 at a university hospital was performed. Patients with knee pain or knee surgery (pre- or post-LSG) were identified using bariatric and orthopedic clinic notes. Those who had improvement in knee pain following LSG resulting in removal from orthopedic surgery waitlist were identified. We also assessed surgical outcomes in patients undergoing knee arthroscopy or total knee arthroplasty (TKA) followed by LSG compared to patients undergoing LSG followed by knee arthroscopy or TKA.

**Results:**

During our study timeframe, 355 patients underwent LSG. Knee pain was documented in 150 (42.2%) patients, and orthopedic surgery consultation was completed for 57 (38.0%) patients with knee pain. Orthopedic intervention was performed prior to LSG for 24 patients and after LSG for 27 patients. Procedures were a combination of arthroscopy (18) and TKA (33). Six patients were removed from the waitlist for TKA following LSG due to resolution of symptoms. Order of interventions did not affect 30-day complications for patients undergoing LSG and arthroscopy (16% arthroscopy first, 0% LSG first, *p* = 0.43). A higher rate of LSG complications was noted in patients who underwent TKA prior to LSG (25% vs 0%, *p* = 0.04). There were no differences in TKA complications (8.3% TKA first, 4.8% LSG first, *p* = 1.00).

**Conclusion:**

In a small number of patients with morbid obesity and severe knee osteoarthritis, orthopedic intervention can be delayed and potentially avoided by undergoing LSG. In our study, 6/57 (10.5%) of patients with orthopedic consultation prior to LSG saw resolution of symptoms of knee pain. Referral to bariatric surgery should be considered for patients with morbid obesity and severe knee osteoarthritis.

## Background

Obesity is a major public health concern in Canada and has a multitude of detrimental health effects including degenerative joint disease^1^. As of 2014, 54% of Canadians self-identified as overweight or obese (61.8% males, 46.2% females)^2^. In Nova Scotia specifically, the rates are higher at 62.6% (68.5% males, 57.1% females)^2^. Evidence suggests that outcomes are worse following joint replacement surgery in overweight or obese individuals^3,4^. Patients with a body mass index (BMI) > 30 are at increased risk of complications including wound infection, bleeding, thrombosis, and longer hospital stays^3^. Patients with a BMI > 50 have been shown to experience a further increase in rates of complications including joint failure and less satisfaction post-operatively when compared to normal weight individuals^3^.

In patients who are morbidly obese and have joint disease requiring surgical intervention, the question remains; which problem should we be addressing first? Conflicting data exists when reviewing outcomes in patients undergoing joint replacement and bariatric surgery. Studies are small and heterogeneous, given the wide range of bariatric procedures performed. A study by Werner et al. showed that morbidly obese patients who had bariatric surgery before TKA had less major and minor complications compared to those who did not have bariatric surgery prior to TKA^4^. In contrast, Nickel et al. showed that patients who had bariatric surgery prior to TKA had higher rates of post-operative complications as well as joint replacement problems including infection, revision, and manipulation when compared to obese patients without bariatric surgery and normal weight individuals. Severson et al.^5^ showed shorter anesthesia and operative times for patients who had bariatric surgery before TKA, but no difference in complication rates and length of hospital stay when compared to patients who underwent TKA before bariatric surgery.

## Methods

This study was approved by the Nova Scotia Health Authority Research Ethics Board (1,022,688), and consent from participants was not required. This was a retrospective cohort study examining patients who underwent sleeve gastrectomy at a single Canadian institution between July 2006 and July 2016. Patients were identified as having knee pain based on clinic notes. For patients with knee pain, we recorded whether or not an orthopedic surgeon was consulted and whether intervention was required (TKA or arthroscopy), either pre- or post-sleeve gastrectomy. If patients underwent an orthopedic intervention, complications were identified and recorded for both the knee surgery and sleeve gastrectomy. The Clavien-Dindo classification was used to rank the severity of the complications. For patients who underwent LSG first, we recorded any improvement in knee pain allowing them to forego an orthopedic procedure. Demographic data including age, gender, weight, height, and BMI (pre- and post- sleeve gastrectomy) were collected for patients with knee pain.

Patients who had both knee surgery and sleeve gastrectomy were compared to determine if type of orthopedic procedure or order of operations significantly impacted outcomes. Chi-square tests were used for categorical variables and Student’s *t* tests were used for continuous variables. Fisher’s exact test was used to compare post-surgery complication rates between subgroups. Binary logistic regression was performed to assess predictors of post-surgery complications. A *p*-value < 0.05 was reported as statistically significant.

## Results

**Fig. 1:**
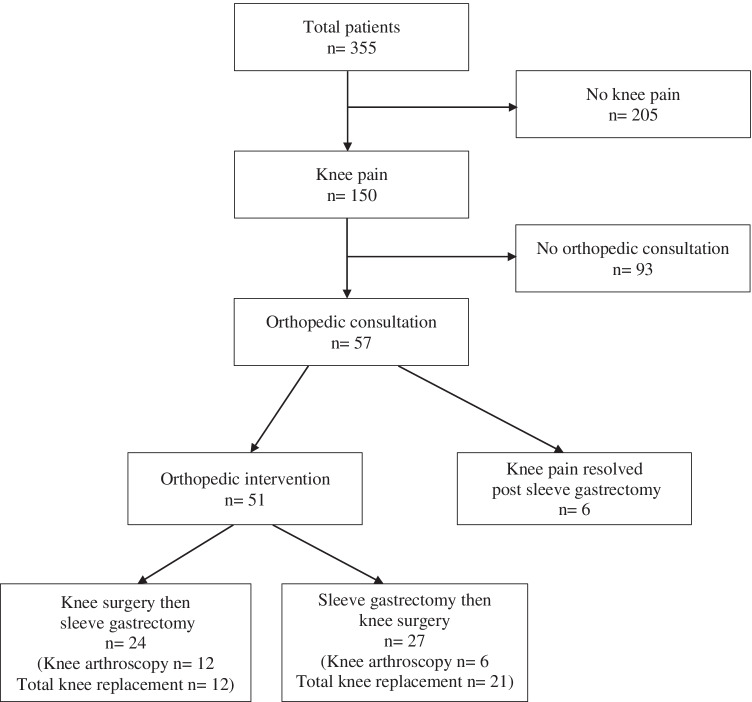
Patient flowchart

A total of 355 patients underwent sleeve gastrectomy from July 2006 to July 2016. Knee pain was noted in 150 patients (42%). Of those, 57 patients had pain requiring consultation to orthopedic surgery. The remaining 93 patients were noted to have knee pain in their pre-operative sleeve gastrectomy consultation only. Fifty-one patients (14%) had both knee surgery and bariatric surgery. Six patients (5 female, 1 male) were identified as having improvement in their knee pain or as no longer requiring orthopedic intervention following bariatric surgery (Fig. 1).

Of the patients with improved knee pain (*n* = 6), two were removed from the waitlist for total knee replacement as their symptoms resolved after sleeve gastrectomy. Both patients were active and able to complete daily activities without issue. A third was diagnosed with osteoarthritis pre-sleeve gastrectomy but, due to young age, was not a candidate for surgery. Following sleeve gastrectomy, symptoms completely resolved. The fourth required multiple corticosteroid injections for osteoarthritis prior to sleeve gastrectomy and had complete resolution of symptoms following bariatric surgery. The fifth was noted to have bilateral knee pain prior to LSG, but orthopedic consultation was not completed until after bariatric surgery. At the time of consultation, symptoms had completely resolved. The final patient ambulated with a cane due to osteoarthritis prior to bariatric surgery and symptoms fully resolved following bariatric surgery. The average weight loss in this group was 84.2 lbs (26–140.8 lbs), and mean reduction in BMI was 12.4 (4.3–24.9). Only one post-operative complication occurred (urinary retention).

Thus, for bariatric surgery patients requiring orthopedic consultation for knee pain, our study shows a 10.5% (6/57) resolution of knee pain following sleeve gastrectomy.

Fifty-one patients had both knee surgery and LSG. The majority were female (71%) and follow up ranged from 1 month to 1 year. The average weight loss was 56.28 pounds (13–147 lbs) with a mean reduction in BMI of 8.88 units (2.5–22.0). A total of 18 patients had knee arthroscopy and LSG; 6 had LSG first. Thirty-three patients had both TKA and LSG; 21 had LSG first.

For patients who had sleeve gastrectomy and arthroscopy, there was no difference in gender, pre-op and post-op BMI, total weight loss, and change in BMI. Patients were significantly older if they had their sleeve gastrectomy first followed by arthroscopy. For patients who had LSG and TKA, patients started with a higher BMI pre-op LSG, if their sleeve gastrectomy was completed first, but also a significantly greater weight loss and change in BMI post-sleeve gastrectomy. Patients were older (59.3 vs 43.2 years old), and the time between both interventions was shorter (3.7 vs 10.4 years) if they had their LSG first. There was no statistical difference between gender or age at LSG (Tables 1 and 2).

**Table 1:** Baseline characteristics of patients who underwent sleeve gastrectomy and arthroscopy

	Total (*n* = 18)	Arthroscopy first (*n* = 12)	LSG first (*n* = 6)	*P*-value
Male gender — *n* (%)	4 (22.2)	2 (16.7)	2 (33.3)	0.423
Age at time of LSG, mean (± SD)	46.4 (9.6)	44.5 (8.2)	50.2 (11.8)	0.250
Pre-op LSG BMI, mean (± SD)	51.4 (9.6)	51.8 (10.3)	50.5 (8.8)	0.790
Post-op LSG BMI, mean (± SD)	41.3 (7.7)	43.2 (8.3)	37.5 (4.9)	0.141
Change in BMI, mean (± SD)	10.1 (6.1)	8.6 (4.9)	13.0 (7.6)	0.154
Total weight loss (lbs), mean (± SD)	66.3 (28.3)	57.3 (28.3)	84.1 (44.0)	0.135
Age at arthroscopy, mean (± SD)	42.3 (14.5)	36.3 (12.1)	54.3 (11.7)	0.008
Years between LSG and arthroscopy, mean (± SD)	6.9 (4.8)	8.3 (5.3)	4.2 (1.9)	0.088

*LSG* laparoscopic sleeve gastrectomy, *BMI* body mass index, *lbs* pounds

**Table 2:** Baseline characteristics of patients who underwent sleeve gastrectomy and total knee arthroplasty

	Entire group (*n* = 33)	TKA first (n = 12)	LSG first (*n* = 21)	*p* value
Male gender — *n* (%)	11 (33.3)	5 (41.7)	6 (28.6)	0.443
Age at time of LSG, mean (± SD)	54.2 (7.2)	53.1 (7.2)	55.0 (7.2)	0.367
Pre-op LSG BMI, mean (± SD)	49.8 (7.9)	43.8 (4.4)	53.2 (7.5)	< 0.001
Post-op LSG BMI, mean (± SD)	41.6 (6.9)	38.4 (5.1)	43.3 (7.2)	0.049
Change in BMI, mean (± SD)	8.2 (4.6)	5.4 (1.6)	9.9 (5.0)	0.005
Total wt loss (lbs), mean (± SD)	50.8 (27.3)	32.5 (8.7)	61.2 (28.9)	0.002
Age at TKA, mean (± SD)	53.2 (11.3)	43.2 (9.4)	59.3 (7.5)	< 0.001
Years between LSG and TKA, mean (± SD)	6.2 (6.9)	10.4 (9.6)	3.7 (3.1)	0.009

*LSG* laparoscopic sleeve gastrectomy, *TKA* total knee arthroplasty, *BMI* body mass index, *lbs* pounds

A total of 7 complications were recorded. Five complications occurred following sleeve gastrectomy, all of which were in patients who underwent knee intervention first. Four had severity scores of 3 (2 post-op bleeds requiring re-operation, 1 sepsis requiring surgical intervention, and 1 patient requiring pacemaker insertion for 2nd degree heart block). One had a severity score of 2 (pulmonary embolism). There was no significant difference in complications between arthroscopy and LSG, regardless of the order of procedure. For patients with TKA and LSG, patients who had knee replacement first were more likely to have a post-operative LSG complications (*p* = 0.040). None of the demographic factors was found to have significant relationship with complications (Tables 3, 4 and 5).

**Table 3:** Proportion of arthroscopy/LSG patients who experienced post-operative complication

	Total (*n* = 18)	Arthroscopy first (*n* = 12)	LSG first (*n* = 6)	*p* value
Post LSG complication — *n* (%)	2 (11.1)	2 (16.7)	0 (0)	0.431
Post arthroscopy complication — *n* (%)	0 (0)	0 (0)	0 (0)	-

*LSG* laparoscopic sleeve gastrectomy

**Table 4:** Proportion of TKA/LSG patients who experienced post-operative complication

	Total (*n* = 33)	TKA first (*n* = 12)	LSG first (*n* = 21)	*p* value
Post LSG complication — *n* (%)	3 (9.1)	3 (25.0)	0 (0)	0.040
Post TKA complication — *n* (%)	2 (6.1)	1 (8.3)	1 (4.8)	1.00

*LSG* laparoscopic sleeve gastrectomy, *TKA* total knee arthroplasty

**Table 5:** Association between post-bariatric surgery complications and demographic factors

Demographic factors	Odds ratio [95% CI]	*p* value
Age at the time of bariatric surgery	1.03 [0.92–1.14]	0.640
Pre-op BMI	0.86 [0.72–1.02]	0.090
Post-op BMI	0.84 [0.69–1.03]	0.100
Change in BMI	0.95 [0.77–1.17]	0.610
Total weight loss (lbs)	0.98 [0.95–1.02]	0.980

Two complications occurred following TKA; one was an operative site infection requiring incision and drainage (TKA followed by LSG group), and the second was a cellulitis treated with IV antibiotics (LSG followed by TKA). No complications occurred following arthroscopy.

## Discussion

Sleeve gastrectomy performed at a multidisciplinary weight loss center is an effective weight loss strategy for morbidly obese patients. Not only does weight loss improve quality of life, but it has been shown to improve co-morbidities; particularly hypertension and diabetes. Obesity is strongly linked to the development of knee osteoarthritis, but the effect of weight loss on osteoarthritis and reducing the need for surgical interventions is less clear^6^. This study looked at the relationship between LSG and knee pain.

In our population, 42% of patients noted knee pain in their pre-operative LSG assessment, and 14% had a surgical intervention completed on their knee either before or after sleeve gastrectomy. We observed that patients who underwent LSG prior to TKA had significantly greater weight loss compared to those who had knee arthroplasty first. This may be secondary to a selection bias given the preoperative BMI in the LSG first group was significantly higher than the TKA first group. As well, there was a shorter time interval between interventions for the LSG first group. There was no difference in post-operative knee complications in those who underwent LSG first. This relationship was not seen when knee arthroscopy was performed but may reflect the severity of osteoarthritis in the LSG first group who had significantly greater BMI.

We noted a statistically significant increase in complications following LSG in patients who had previously underwent TKA. However, there was no difference in complications regardless of procedure order for knee arthroscopy. A study by Severson et al.^5^ in 2012 compared operative outcomes between patients who underwent TKA and bariatric surgery and did not find a difference in complication rates between patients who had knee replacement before bariatric surgery, < 2 years after bariatric surgery, and > 2 years after bariatric surgery. Those who had bariatric surgery followed by knee surgery within 2 years had the lowest rates of complications, but sample size was small. Our study also seems to be in line with this final finding. A study by Parvizi et al.^7^ looked at 20 patients who were treated with bariatric surgery before total hip or knee replacement. They found that joint function and symptoms significantly improved after weight loss surgery, and no issues with quality of the joint replacement was seen with the pre-operative weight loss. Important to note, in this study, the time between operations is long, so it is unclear if there is a correlation between procedures or if the results are due to an unknown variable or small sample size.

This retrospective chart review aimed to identify patients who deferred or no longer required knee surgery after sleeve gastrectomy and to gain a better understanding of the relationship between obesity and knee pain. The data gathered shows that it is possible to have resolution of knee pain with weight loss as this occurred in a small cohort of our patients (*n* = 6). Not only does this reduce the risk of undergoing a second surgical procedure and the complications associated, but this is locally important as the average wait times for both bariatric and joint replacement is long. The average wait time for knee replacement is currently 190 days for a consultation and 256 days for surgery. Ninety percent of patients have their knee replaced within 617 days^8^. This is much higher than the CIHI benchmark goal of 90% receiving knee replacement within 182 days^9^. If joint replacement need could be reduced by providing bariatric surgery to those who qualify, it could help with decreasing the burden of wait times in the area. These patients had a high mean reduction in BMI 12.4 (4.3–24.9). This may indicate that patients with higher BMI stand to benefit the most from the LSG first approach in terms of resolution of knee pain without need for operative intervention.

This study has several limitations. The first being the nature of the retrospective chart review and the inherent risk of missing data, such as timing of resolution of the six patients’ knee pain and possible confounding factors for post-operative complications. Data was gathered through multiple sources including two electronic data bases and paper charts. This would not capture patients visiting their primary care physician for post-operative issues. Our population includes patients outside of the electronic database catchment area and orthopedic clinic visits would not be available for those patients seen out of province. This may result in underestimating complications for all procedures and the number of patients requiring orthopedic procedures following weight loss surgery. Second, follow up times at the bariatric clinic varied widely between patients from 1 month to 1 year. We used the last documented weights and BMIs from these clinic visits, but if follow-up was short then final weight loss achievements would not have been captured. Third, the sample size is small, and it is difficult to draw conclusions based on the findings, particularly due to the low number of complications. As well, the long wait times between surgeries limits our ability to assert tighter conclusions. There is a significant discrepancy in Nova Scotia between wait times for TKA and LSG. Ninety percent of patients receive their TKA within 617 days compared to 180 days for LSG. Numerous factors are likely the cause of this including resources, access to care, and comorbidity profile of our patients. Fourth, we did not include complication rates for a control group of patients who underwent LSG alone or TKA alone, so we cannot say that complication rates in the studied cohort are different. Lastly, other bariatric procedures such as gastric bypass and duodenal switch were not analyzed as the study center primarily performs LSGs. Future studies can assess the effect of these other bariatric procedures on their effect on knee pain resolution for bariatric surgery patients requiring concurrent orthopedic consultation.

## Conclusion

Obesity and knee pain commonly occur together, and management can be challenging due to a multitude of factors. Our work has demonstrated that patients can have significant improvement in knee symptoms following sleeve gastrectomy, allowing a small proportion of them to defer or forego orthopedic surgery previously deemed necessary.

## Acronyms

LSG: Laparoscopic sleeve gastrectomy
TKA: Total knee arthroplasty
BMI: Body mass index
